# NeoAgDT: optimization of personal neoantigen vaccine composition by digital twin simulation of a cancer cell population

**DOI:** 10.1093/bioinformatics/btae205

**Published:** 2024-04-13

**Authors:** Anja Mösch, Filippo Grazioli, Pierre Machart, Brandon Malone

**Affiliations:** Biomedical AI Group, NEC Laboratories Europe GmbH, Heidelberg 69115, Germany; Biomedical AI Group, NEC Laboratories Europe GmbH, Heidelberg 69115, Germany; Biomedical AI Group, NEC Laboratories Europe GmbH, Heidelberg 69115, Germany; Biomedical AI Group, NEC Laboratories Europe GmbH, Heidelberg 69115, Germany

## Abstract

**Motivation:**

Neoantigen vaccines make use of tumor-specific mutations to enable the patient’s immune system to recognize and eliminate cancer. Selecting vaccine elements, however, is a complex task which needs to take into account not only the underlying antigen presentation pathway but also tumor heterogeneity.

**Results:**

Here, we present NeoAgDT, a two-step approach consisting of: (i) simulating individual cancer cells to create a digital twin of the patient’s tumor cell population and (ii) optimizing the vaccine composition by integer linear programming based on this digital twin. NeoAgDT shows improved selection of experimentally validated neoantigens over ranking-based approaches in a study of seven patients.

**Availability and implementation:**

The NeoAgDT code is published on Github: https://github.com/nec-research/neoagdt.

## 1 Introduction

Harnessing a patient’s own immune system to fight tumors has become a successful treatment option for cancer. Tumor-specific mutations, which can be detected as foreign by the immune system, tend to accumulate in cancers, and their exploitation as a therapeutic target has been proven to work for a multitude of tumor types (van den Berg *et al.* 2020, Creelan *et al.* 2021, Kristensen *et al.* 2022). For this purpose, vaccines that stimulate the patient’s T cells to recognize immunogenic mutations, so-called *neoantigens*, have been shown to be an efficient and reliable method (Ott *et al.* 2017, Keskin *et al.* 2019, Cafri *et al.* 2020).

More specifically, T cells recognize altered peptides resulting from tumor-specific mutations that change a protein’s amino acid sequence. These peptides are presented on the surface of tumor cells by major histocompatibility complex (MHC) class I and II, also referred to as human leucocyte antigen (HLA) in humans. Cytotoxic T cells bind to these peptides and induce cell death, hence killing the tumor cells. The mutational landscape of a tumor can be extremely heterogeneous (Alexandrov *et al.* 2020), so different cancer cells from the same tumor often present different neoantigens (Jia *et al.* 2018, Łuksza *et al.* 2022). This diversity makes it difficult to select target neoantigens for vaccination or other anti-tumor treatments, and a multitude of criteria have been used to filter for the most promising candidates (Mösch *et al.* 2019, Richters *et al.* 2019). Current practices suggest applying prediction methods to several steps of the antigen presentation pathway, including peptide processing and MHC binding. Together with gene expression, allele frequency of the mutation, and other features, rankings are created based on these criteria; the top candidates go into the vaccine manufacturing process (Richters *et al.* 2019).

Many different neoantigen prediction tools aim to provide a user-friendly experience and try to facilitate the process of identifying suitable neoantigen targets for therapeutic purposes (Bais *et al.* 2017, Kim *et al.* 2018, Hasegawa *et al.* 2020, Hundal *et al.* 2020, Rieder *et al.* 2022). These tools, such as Neopepsee, are usually designed as an all-in-one pipeline starting with raw sequencing data; they typically use a variety of algorithms for different aspects of the antigen presentation pathway from variant calling to HLA typing and MHC binding prediction, but also additional immunogenicity features.

However, the decision on including a putative neoantigen into a vaccine based on ranking predictions or other fixed computational numbers represents a complex task which requires experience to identify suitable cut-off or threshold values. Such selection processes also carry the risk of excluding a promising target that does not pass the cut-off on one category of ranking.

**Figure 1.**
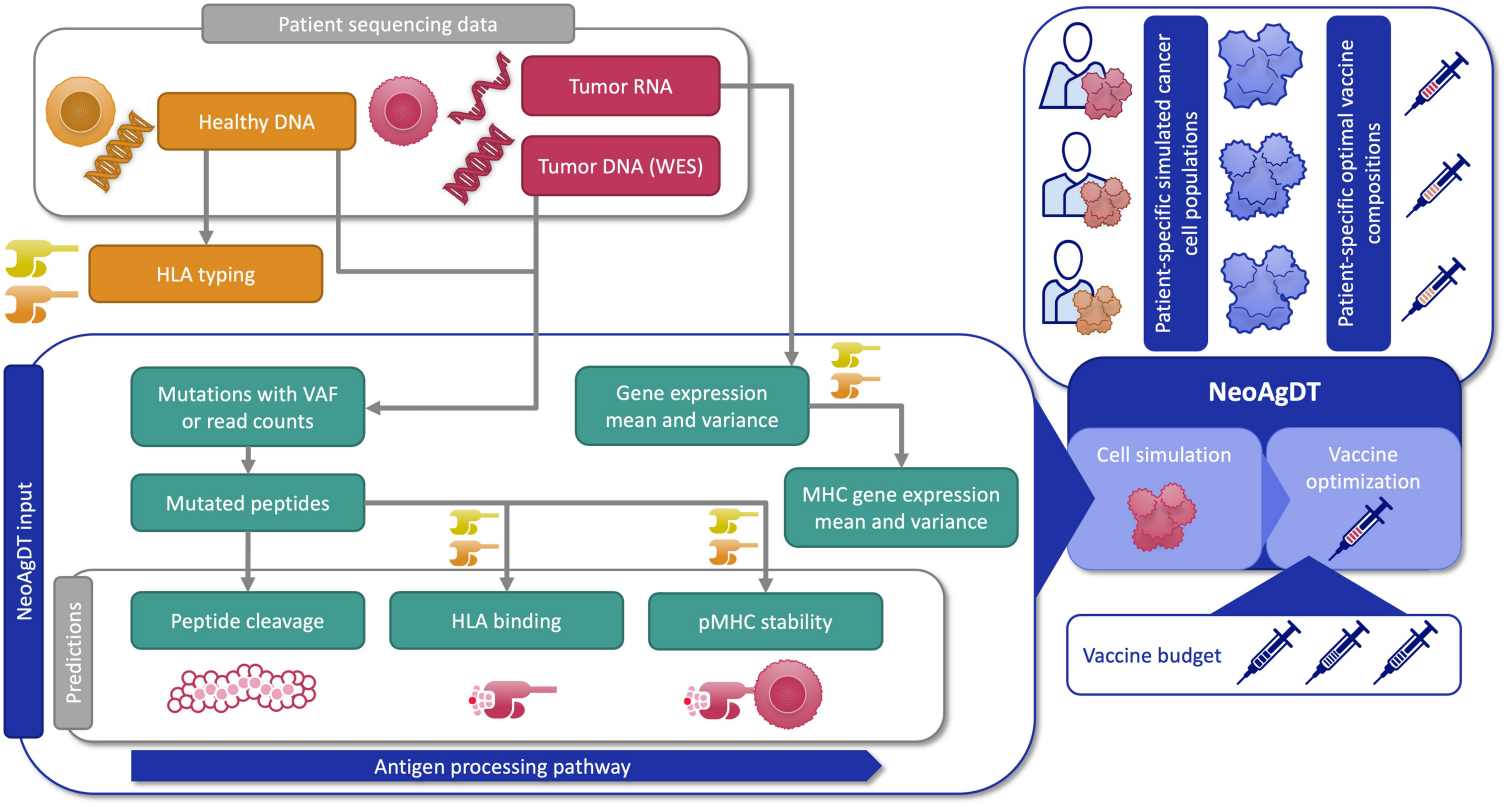
Workflow of NeoAgDT from sequencing data to vaccine composition. Variant/mutation calling and gene expression steps are not part of the pipeline but are shown for general understanding of data flow. NeoAgDT input data are shown as individual boxes (green) in the large box at the bottom left. For this publication, pVACtools (Hundal *et al.* 2020) is used for the predictions, which are indicated by a separate box containing the three related green boxes. However, it is also possible to use other tools.

Here, we present an improved method to determine the optimal vaccine composition, i.e. the neoantigens that should be included as elements in a vaccine. Our approach consists of two parts. First, a cell population of the patient’s tumor is created by simulating antigen presentation pathway steps based on presence and expression of a mutation for each individual cell. Each of these steps can be represented by a suitable prediction from a pipeline or a method. In this work, we use pVACtools (Hundal *et al.* 2020), but using other pipelines or multiple tools for different steps is also possible. Second, the optimal vaccine composition is computed by maximizing the likelihood of immune response in the simulated cancer cell population. This approach also takes into account a budget constraint on the number of elements to be included in the vaccine; the budget depends on the vaccine manufacturing process (see Fig. 1). We call this approach *NeoAgDT* (Neoantigen Digital Twin) since it creates a digital twin of the patient’s tumor. Digital twins originally come from industrial applications but are gaining more and more traction for health care applications as more data becomes available and deeper understanding of underlying biological processes increases (Malone *et al.* 2020, Hernandez-Boussard *et al.* 2021, Laubenbacher *et al.* 2022, Stahlberg *et al.* 2022).

We demonstrate the application of NeoAgDT on a dataset of seven patients with gastrointestinal cancers (Tran *et al.* 2015), for which T cell assays confirmed immunogenicity of some mutations. We show that NeoAgDT improves recall compared to eleven traditional ranking-based approaches relying on MHC binding prediction thresholds. Therefore, vaccines created with NeoAgDT are more likely to elicit an immune response by the patient in the presented example cases.

## 2 Materials and methods

### 2.1 Data

We used sequencing and mutation data from Tran *et al.* (2015) to validate NeoAgDT. Tran *et al.* (2015) performed T cell assays to confirm the immunogenicity of tumor-specific mutations; these assay results allow us to verify if NeoAgDT captures these neoantigens in a proposed vaccine composition. From nine patients whose tumors were sequenced, seven could be used for our experiments. Sample 4069 was not considered because it only contains 25 mutations that could all be included in a vaccine, so that no optimization of the composition is needed. For Sample 3971, no variant allele frequency information is available.

### 2.2 Pre-processing

RNA sequencing data was downloaded from the Sequence Read Archive (SRA) available under the accession number PRJNA342632. RNA reads were mapped to the human genome reference hg19 as in the original publication. We applied STAR version 2.7.10a with default parameters (Dobin *et al.* 2013) using the gene annotation of gencode v41 mapped to hg37/hg19 (gtf). Expression was calculated using cufflinks version 2.2.1 with default parameters (Trapnell *et al.* 2010).

Mutations and mutation frequency were taken from the supplementary materials of Tran *et al.* (2015). For peptide processing, binding, and stability prediction, pVACbind of pVACtools version 3.0 (Hundal *et al.* 2020) was used. Mutated peptide sequences were derived from tandem minigene sequences given in the supplementary materials of Tran *et al.* (2015). For compatibility with NeoAgDT, these sequences were converted to FASTA format. pVACbind was run with standard parameters, and NetMHCpan 4.1 prediction percentiles were used for MHC binding affinity (Reynisson *et al.* 2020). NetChop was run with –method 20 s (Keşmir *et al.* 2002) and –threshold 0.0 parameters, and NetMHCstab (Rasmussen *et al.* 2016) was used as downstream analysis of the all_epitopes.tsv output file generated by pVACbind. As a result, MHC binding prediction, cleavage prediction, and MHC stability prediction are available for every possible mutated peptide. Additional processing of the data was performed to prepare inputs that are suitable for the cell simulation, including gene identifier mapping to Ensembl ID, calculating gene expression variance from confidence intervals, and creation of an HLA allele-specific expression table. A Jupyter notebook containing these pre-processing steps is included in the NeoAgDT repository.

To compare NeoAgDT to threshold-based binding predictions, six methods from the pVACbind output were considered, namely NetMHC (Andreatta and Nielsen 2016), NetMHCpan (Reynisson *et al.* 2020), NetMHCcons (Karosiene *et al.* 2012), MHCflurry (O’Donnell *et al.* 2018), MHCnuggetsI (Shao *et al.* 2020), and PickPocket (Zhang *et al.* 2009).

### 2.3 Cell simulation

To select the optimal vaccine composition, individual cancer cells are simulated to provide a population of size *M*. In order to model statistical variability, this process is repeated *K* times, leading to multiple populations of cells. *K* and *M* are parameters which can be set by the user. We generate a cell population with a probabilistic simulation constituted by a sequence of sampling steps aimed at determining the peptide-MHC (pMHC) complexes presented on each individual cell surface.

#### 2.3.1 Assertion of mutation presence

For a given patient, let $N={\left\{v_{i}\right\}}_{i=1}^{N}$ be the set of variants, i.e. tumor-specific mutations. Let $C={\left\{c_{j}\right\}}_{j=1}^{M}$ be the set of cancer cells that we aim to simulate. For each cell, we model the presence of a variant $v_{i}$ by means of a random variable $V_{i}$ that follows a Bernoulli distribution determined by the variant allele frequency (VAF). For a given variant, VAF is calculated by dividing the number of DNA whole exome sequencing (WES) reads $d_{v_{i}}$ containing that variant ($d_{v_{i}}^{\mathrm{ALT}}$) by the number of all reads covering the given position ($d_{v_{i}}^{\mathrm{REF}}\;+\;d_{v_{i}}^{\mathrm{ALT}}$):

(1) $$V_{i}∼\mathrm{Bernoulli}\left(\frac{d_{v_{i}}^{\mathrm{ALT}}}{d_{v_{i}}^{\mathrm{REF}}\;+\;d_{v_{i}}^{\mathrm{ALT}}}\right)\;.$$

For each variant, if the sampling from the distribution defined by Equation (1) results in a positive outcome, the following steps will take place.

#### 2.3.2 Estimation of protein abundance

Protein abundance is calculated from gene expression data assuming a direct relation from gene to protein expression (Schwanhäusser *et al.* 2011). For each cell, if variant $v_{i}$ was sampled to be present in the previous step, let us consider its corresponding gene $g_{i}$. Gene transcript abundance is modeled as a random variable $\lambda_{g_{i}}$ that follows a Gamma-Poisson distribution parameterized by the empirical mean gene expression $\bar{g_{i}}$ and variance $\mathrm{Var}(g_{i})$:

(2) $$\lambda_{g_{i}}∼\mathrm{Gamma}-\mathrm{Poisson}(\bar{g_{i}},\mathrm{Var}(g_{i}))\;.$$

The number of proteins that actually carry variant $v_{i}$ is then calculated by multiplying the gene transcript abundance $\lambda_{g_{i}}$ by the VAF calculated from RNA reads $r_{v_{i}}$:

(3) $$n_{v_{i}}^{\mathrm{prot}}=\lambda_{g_{i}}\cdot \frac{r_{v_{i}}^{\mathrm{ALT}}}{r_{v_{i}}^{\mathrm{REF}}+r_{v_{i}}^{\mathrm{ALT}}}\;.$$

Analogously to Equation (2), for each HLA allele *a*, we obtain

(4) $$n_{a}^{\mathrm{MHC}}∼\mathrm{Gamma}-\mathrm{Poisson}(\bar{g_{a}},\mathrm{Var}(g_{a}))\;,$$

where $\bar{g_{a}}$ is the mean and $\mathrm{Var}(g_{a})$ is the variance of the gene expression of gene $g_{a}$ associated to the allele. To compute the number of MHC molecules, we assume a unitary VAF ratio. In our experiments, we restrict the analysis to MHC class I molecules.

#### 2.3.3 Cleavage of protein into peptides

For each variant $v_{i}$, let $P_{i}={\left\{p_{k}^{i}\right\}}_{k=1}^{O^{i}}$ be the set of minimal epitopes (peptides) which can be cleaved from its associated protein. We obtain these epitopes, 8- to 11-mers containing at least one altered amino acid at any position, from pVACbind output. For our experiments, we compute the vector $c_{i}\in R^{O^{i}}$ constituted by the cleavage scores predicted by NetChop for each resulting peptide. However, our cell simulation supports the adoption of any other predictive model for cleavage.

In each simulated cell, for a given protein indexed by *i*, the count of each associated peptide is drawn according to a multinomial distribution parameterized by the number of trials $n_{v_{i}}^{\mathrm{prot}}$ and the event probability vector $\frac{c_{i}}{||c_{i}|{|}_{1}}$:

(5) $$m_{i}∼\mathrm{Multinomial}(n_{v_{i}}^{\mathrm{prot}},\frac{c_{i}}{||c_{i}|{|}_{1}})\;.$$

Then, we define the corresponding multiset $P_{i}^{*}$ of peptides with multiplicity:

(6) $$P_{i}^{*}=(P_{i},m_{i})\;,$$

where $P_{i}$ is the underlying set of the multiset, formed by its distinct elements, and $m_{i}\in Z^{O^{i}}$ is a vector of integers which define the multiplicity of the underlying set elements. That is, $P_{i}$ gives the count of each cleaved peptide derived from $v_{i}$. For a given cell, considering all available variants, the multiset of all cleaved peptides is given by

(7) $$P^{*}=\underset{\begin{array}{c} 1\leq i\leq N\\ \text{if}\;V_{i}=1 \end{array}}{\cup }P_{i}^{*}.$$

#### 2.3.4 Binding of peptide to available MHC molecules

In this section, we model the competition for available MHCs to which the peptides can bind. For a given patient, let us consider the set A of its HLA alleles *a*. For a given cell, the binding affinity value for each HLA allele *a* is stored in vector $ba_{a}\in R^{\left|P^{*}\right|}$ of all peptides in $P^{*}$ with *a*. For our experiments, we use NetMHCpan 4.1 as part of pVACbind to predict these values but any other prediction method or experimentally measured affinity can be used. We treat the predicted binding affinities as proportional to the binding events. Similarly to the cleavage in the previous subsection, the number of peptides binding with allele *a* is drawn according to the following multinomial distribution:

(8) $$m_{a}∼\mathrm{Multinomial}(n_{a}^{*},\frac{ba_{a}}{||ba_{a}|{|}_{1}})\;,$$

where $n_{a}^{*}=\min(n_{a}^{\mathrm{MHC}},|P^{*}|)$. Then, given a multiset of peptides $P^{*}$ defined previously, we define the corresponding multiset $B_{a}^{*}$ of pMHC complexes formed by all peptides in $P^{*}$ and a given allele *a*, with multiplicity $m_{a}$. Finally the multiset $B^{*}$ of binding pMHC complexes for all alleles is obtained as:

(9) $$B^{*}=\underset{a\in A}{\cup }B_{a}^{*}\;.$$

This methodology ensures that the final pool of pMHC complexes is enriched for peptides that have higher binding affinities to their corresponding MHC.

#### 2.3.5 Presentation of available pMHC complexes

The final step of the simulation is the modeling of pMHC complex presentation on the cell surface. For each cell, we use predicted stability scores for each bound pMHC complex in $B^{*}$ and obtain the vector $st\in R^{\left|B^{*}\right|}$. In our experiments, we use NetMHCstab to predict such scores. The presentation of the pMHC complexes is obtained via sampling from a binomial distribution:

(10) $$m∼\mathrm{Binomial}(1,\frac{st}{||st|{|}_{1}})\;.$$

We then denote by $S^{*}$ the multiset of all pMHC complexes presented on the surface of a given cell from the multiset of binding complexes $B^{*}$ with multiplicity m.

### 2.4 Aggregating minimal epitopes

A vaccine element $v_{i}$ is a putative neoantigen, i.e. a mutation centered in a long amino acid chain (e.g. 27-mers). Within cancer cells, these chains of amino acids are chopped into smaller peptides (e.g. 8-, 9-, 10-mers). These smaller peptides—also known as epitopes, or neoepitopes when derived from neoantigens—can be presented on the surface of the cancer cells. This only happens if the peptide successfully binds to a major histocompatibility complex (MHC) molecule. We can formally represent a vaccine element $v_{i}$ as a set of $O^{i}$ minimal epitopes: $v_{i}={\left\{p_{k}^{i}\right\}}_{k=1}^{O^{i}}$.

### 2.5 Vaccine optimization

After simulating the pMHC complexes on the surface of each cell resulting in a list of epitopes and their associated mutations, we turn to the problem of selecting the candidate elements to include in the vaccine design. The aim of this step is to select a set of candidate elements to include in the vaccine which will maximize the number of cells covered by the vaccine. The size of the set is restricted by the vaccine budget, i.e. the number of vaccine elements that can be included in the vaccine modality of interest.

Let E⊂N be an arbitrary subset of N defined by the set of integer (Boolean) selectors $X={\left\{x_{i}\right\}}_{i=1}^{N}$, where

(11) $$\begin{array}{c} x_{i}=\{\begin{array}{cc} 1, & \text{if}\;e_{i}\in E.\\ 0, & \text{if}\;e_{i}\notin E. \end{array} \end{array}$$

E consists of the selected vaccine elements, i.e. neoantigens, which constitute the vaccine. Our objective is to find the optimal set E which maximizes the likelihood of having immune response for all cancer cells in C. This means that our objective is:

(12) $$\underset{E}{\max}P(R=+|E,C)\;\;\;,$$

where R=+ represents positive immune response.

Maximising immune response might have different interpretations; hence, we introduce two different objectives with slightly different interpretations.

#### 2.5.1 Kill the maximum number of cells

We define

(13) $$\underset{E}{\max}P(R=+|E,C):=\underset{E}{\min}P(R=-|E,C)\;\;\;.$$

By modeling the probability of having no immune response in all cells as the joint probability of having no immune response in each cell, and assuming conditional independence, we can write

(14) $$P(R=-|E,C)=\prod_{j=1}^{M}P(R=-|E,c_{j})\;\;\;.$$

We consider that a vaccine E causes a positive response if at least one of its elements $e_{i}$ causes a positive response. That is, for a given cell $c_{j}$, the probability of no response $P(R=-|E,c_{j})$ is the joint likelihood that all elements fail:

(15) $$P(R=-|E,c_{j})=\prod_{i=1}^{N}P(R=-|e_{i},c_{j})x_{i}\;,$$

where $x_{i}$ is the selector of the *i*th vaccine element $e_{i}$ and defines its inclusion in the vaccine. It follows that

(16) $$P(R=-|E,C)=\prod_{j=1}^{M}\prod_{i=1}^{N}P(R=-|e_{i},c_{j})x_{i}\;.$$

We define the log-probability of no immune response for a cell $c_{j}$ by including vaccine element $e_{i}$ in the vaccine as

(17) $$p_{ij}:=\mathrm{logP}(R=-|e_{i},c_{j})\;.$$

Since the logarithm is monotonic, the objective of Equation (13) can be rewritten as

(18) $$\underset{E}{\min}\sum_{j=1}^{M}\sum_{i=1}^{N}p_{ij}x_{i}\;.$$

We refer to the objective of Equation (18) as MinSum.

For each vaccine element $e_{i}$ we define a cost $k_{i}$, which can either be constant or a function of its peptide length. We constrain Objective 18 in the following way:

(19) $$\sum_{i=1}^{N}k_{i}x_{i}\leq b\;.$$

**Figure 2.**
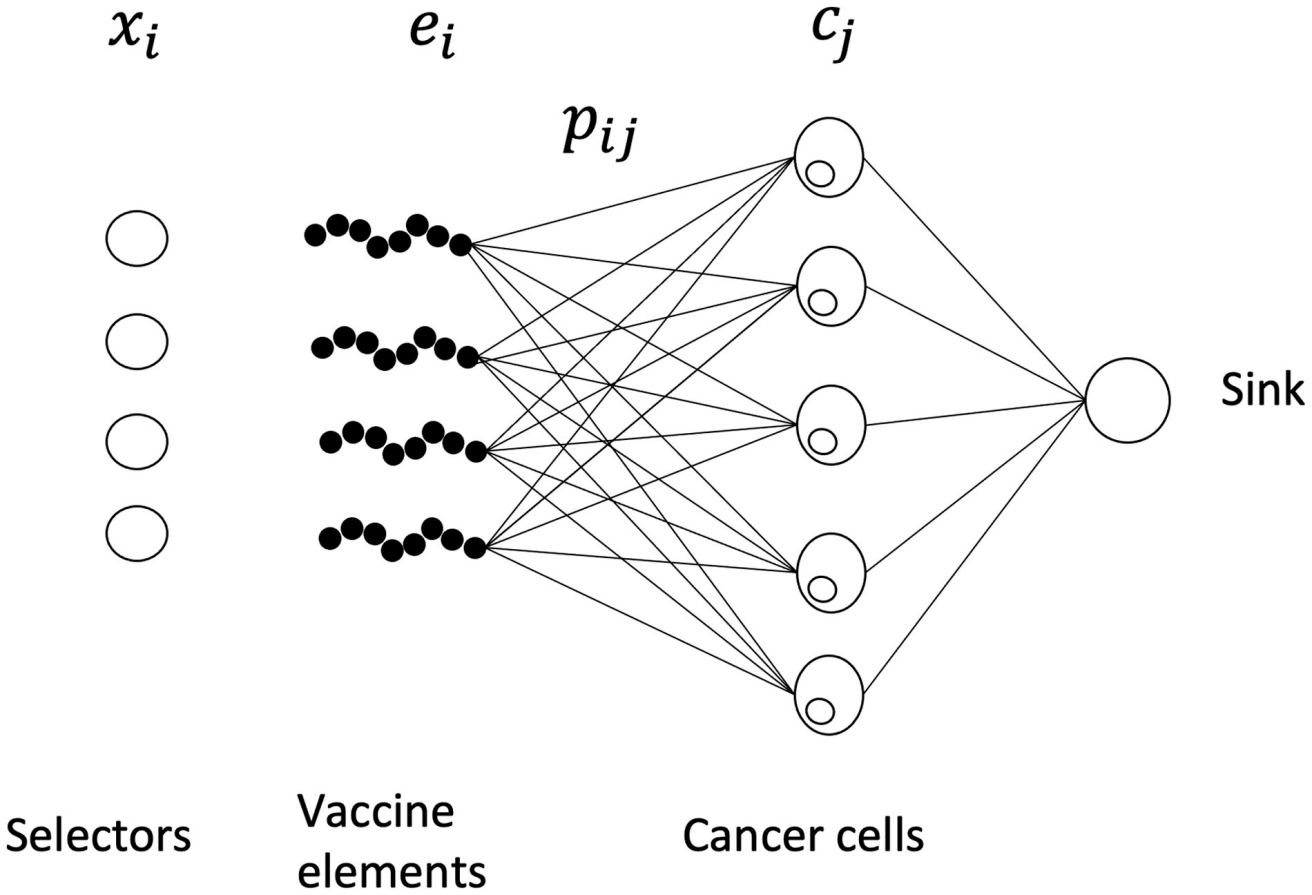
Representation of MinSum optimization, Equation (18) as a network flow problem.

We approach this problem as a type of network flow problem (Cormen *et al.* 2022) (see Fig. 2), with one set of nodes corresponding to vaccine elements, one set corresponding to cancer cells, and one sink. The goal is to select the set of vaccine elements such that the likelihood of no response across the whole population of cells is minimized.

We can optimize Equation (18) subject to the constraint of Equation (19) through integer linear programming (ILP) (Cormen *et al.* 2022).

#### 2.5.2 Maximize the likelihood to kill the most resistant cell

In this section, we introduce a slightly different optimization problem. Although the same formalization of the problem still holds, here we define

(20) $$\underset{E}{\max}P(R=+|E,C):=\underset{E}{\min}\underset{c\in C}{\max}\{P(R=-|E,c)\}\;.$$

In this setting, we approach vaccine design by minimizing the probability of no response for the cell which has the highest probability of no response.

From Equations (17) and (15) we derive

(21) $$\underset{E}{\min}\underset{1\leq j\leq M}{\max}\sum_{i=1}^{N}p_{ij}x_{i},$$

subject to the constraint of Equation (19). We refer to the objective of Equation (21) as MinMax.

Standard ILP solvers cannot directly solve this minimax problem; however, we use the standard approach of a set of surrogate variables to address this problem. In particular, we define $x_{j}^{c}$ to be the log-likelihood of no response for cell $c_{j}$. That is, $x_{j}^{c}=\sum_{i=1}^{N}l\mathrm{ogP}(R=-|e_{i},c_{j})x_{i}$. Further, we define

(22) $$z:=\underset{1\leq j\leq M}{\max}x_{j}^{c}\;\;;$$

that is, *z* is the maximum log-likelihood that any cell does not respond to the vaccine (or, alternatively, the minimum log-likelihood that any cell will respond to the vaccine). Finally, then, our aim is to minimize *z*:

(23) $$\underset{E}{\min}z\;\;.$$

**Figure 3.**
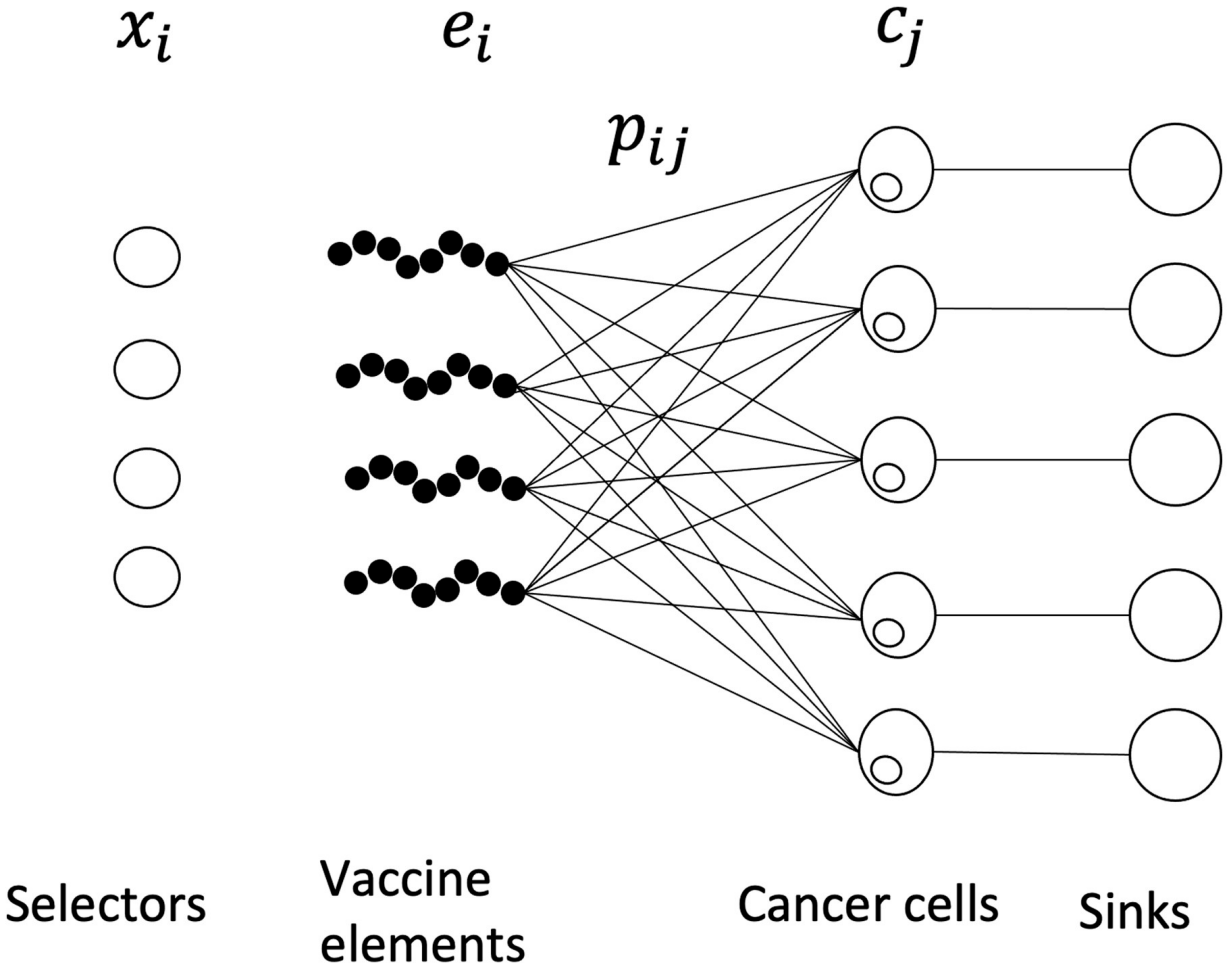
MinMax optimization as a network flow problem. Representation of the optimization of Equation (21) as a network flow problem.

Our problem is essentially a min-flow problem with multiple sinks, where each cell is a sink. However, our aim is to minimize the flow to each individual sink rather than the flow to all sinks (see Fig. 3). In particular, rather than the “sum” operator typically used to transform multiple-sink flow problems into a single-sink problem, we would need a (nonlinear) “min” operator. Thus, efficient min-flow formulations are not applicable in this setting.

In our approach, the probabilistic cell simulation simulates transcription, translation and the antigen processing pathway. Each simulated cell, indexed by *j*, can be formally represented by the multiset of peptides presented on its surface, what we also refer to as minimal epitopes, $S_{j}^{*}$ as defined in Section 2.3.5.

We consider that a vaccine element $e_{i}$ causes a positive response if at least one of its associated minimal epitopes $p_{k}^{i}$ causes a positive response. Hence, we can express the probability of no response for a given cell indexed by *j* and presenting a multiset of epitopes $S_{j}^{*}$, conditioned on vaccine element $e_{i}$, as

(24) $$P(R=-|e_{i},S_{j}^{*})=\prod_{k=1}^{O^{i}}P(R=-|p_{k}^{i},S_{j}^{*})\;\;.$$

In light of this, we express the $p_{ij}$ coefficients associated with vaccine element $e_{i}$ and the cell indexed by *j* [see Equation (17)] as follows:

(25) $$p_{ij}:=\mathrm{logP}(R=-|e_{i},S_{j}^{*})=\log\prod_{k=1}^{O^{i}}P(R=-|p_{k}^{i},S_{j}^{*})\;\;.$$

Finally, for a given minimal epitope $p_{k}^{i}\in e_{i}$, we model its probability of inducing immune response in the cell indexed by *j* as

(26) $$P(R=-|p_{k}^{i},S_{j}^{*})=\max(0,1-\lambda N_{jk}^{i})\mathrm{DFS}(p_{k}^{i})\;\;,$$

where $N_{jk}^{i}$ is the number of times $p_{k}^{i}\in e_{i}$ is presented by the cell indexed by *j*, λ is a hyper-parameter, and DFS:Peptide→[0,1] is a function which models distance-from-self in order to approximate T cell recognition. In our experiments, we set $\lambda ={\left(\max N_{jk}^{i}\right)}^{-1}$ across all simulated cells in order to avoid a saturation effect. This guarantees that only the most often presented peptide leads to a 100% response probability for the cell that presents it the maximum number of times.

From Equations (25) and (26), we formulate the $p_{ij}$ coefficients as

(27) $$p_{ij}=\log\prod_{k=1}^{O^{i}}max(0,1-\lambda N_{jk}^{i})\mathrm{DFS}(p_{k}^{i})\;.$$

## 3 Results

### 3.1 Vaccine composition

We compared the recall of neoantigens confirmed by Tran *et al.* (2015) in NeoAgDT vaccine element compositions with eleven threshold-based standard approaches of vaccine elements selected by using six commonly used binding prediction that are part of pVACbind. For each method, both binding affinity scores and ranking were considered, if available. These prediction results were generated as part of the pVACbind output used for the cell simulations. For NeoAgDT, results are averaged over 10 repeated simulations for populations of 1000 cancer cells. Standard errors are omitted because all 10 simulations achieved the same scores. For both ranking approaches, scores and percentiles, the top 10 vaccine element candidates were considered; NeoAgDT was also given a vaccine budget of 10. All candidates selected also meet the standard threshold of ≤500 nM for binding scores and ≤2.0 for percentiles. Results for neoantigens recognized by CD8^+^ T cells are shown in Table 1.

### 3.2 Response probability analysis

One of the main advantages of the NeoAgDT approach is the possibility to estimate a response probability, i.e. the likelihood of a simulated cancer cell to get eliminated by a CD8^+^ T cell, for a given vaccine composition. In particular, after selecting an optimal vaccine design, Equation (15) allows us to compute a response probability for each cell.

**Figure 4.**
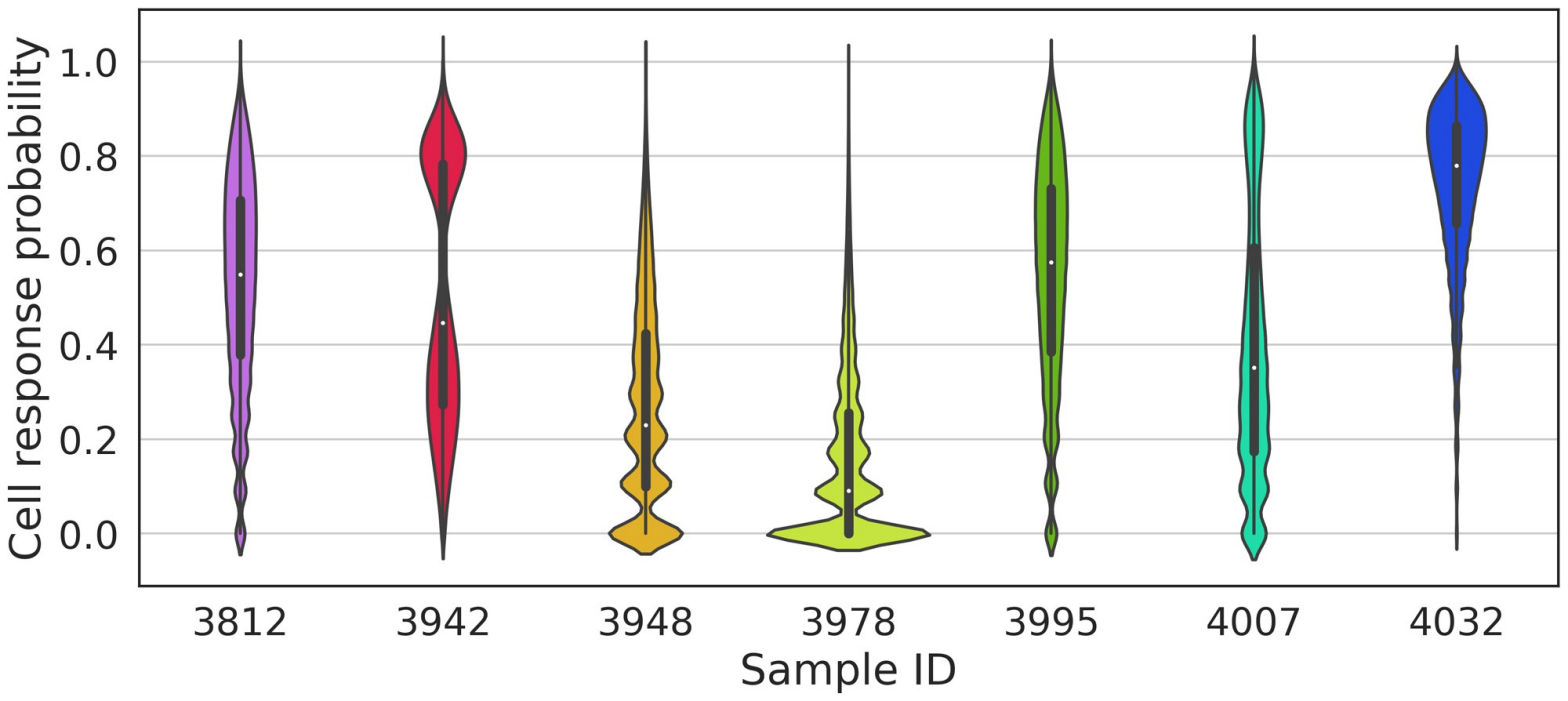
Distributions of the response probabilities for all simulated cells, given the optimal vaccine composition. The population size has been set to 10 000 and the MinSum objective has been optimized. For each patient, a total of 10 cell populations are considered.

Figure 4 depicts the distributions of the response probabilities for all considered patients. As shown, we observe that for some patients the optimal vaccine composition guarantees high response. However, we also observe patients, e.g. 3978, for which the computed optimal vaccine still results in mostly low response probabilities.

This feature of our system is particularly helpful for providing insights on the estimated efficacy of a vaccine. This information is normally not provided by other methodologies since they only rank neoantigens; in contrast, NeoAgDT simulates cancer cells and their set of presented peptides, so it inherently provides such efficacy estimates.

**Figure 5.**
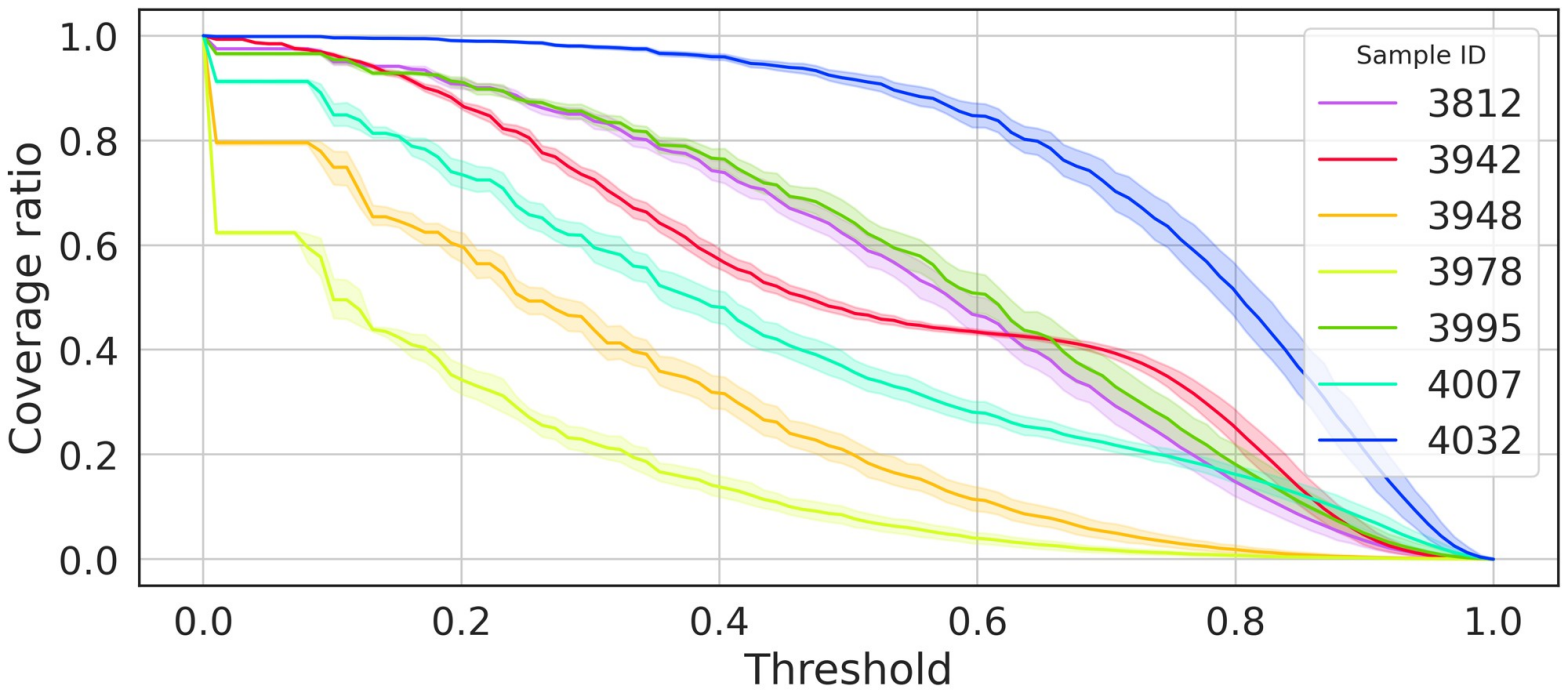
Coverage ratio refers to the proportion of responding cells at the response probability threshold indicated on the *x*-axis. The population size has been set to 10 000 and the MinSum objective has been optimized for computing the vaccine composition. Confidence intervals are over 10 repeated simulations.

Estimating a response probability for each simulated cell additionally allows NeoAgDT to count how many cells have a response probability higher than a given threshold. Figure 5 depicts the fraction of cells expected to respond to the optimal vaccine design as we vary the response probability threshold.

**Table 1.** Comparison of vaccine validation results for CD8 restricted neoantigens computed by considering the validated mutations from Tran *et al.* (2015) as ground truth.^a^

Sample	VM	NeoAgDT			NetMHC Percentile			NetMHCpan Perc.			NetMHCcons Perc.			MHCflurry Perc.			PickPocket Perc.		
		TP	FN	Recall	TP	FN	Recall	TP	FN	Recall	TP	FN	Recall	TP	FN	Recall	TP	FN	Recall
3812	0	^b^			^b^			^b^			^b^			^b^			^b^		
3942	2	**1**	**1**	**0.50**	0	2	nan	**1**	**1**	**0.50**	0	2	nan	**1**	**1**	**0.50**	0	2	nan
3948	0	^b^			^b^			^b^			^b^			^b^			^b^		
3978	0	^b^			^b^			^b^			^b^			^b^			^b^		
3995	3	2	1	0.67	2	1	0.67	2	1	0.67	1	2	0.34	1	2	0.34	1	2	0.34
4007	2	**2**	**0**	**1.00**	**2**	**0**	**1.00**	**2**	**0**	**1.00**	**2**	**0**	**1.00**	**2**	**0**	**1.00**	0	2	nan
4032	3	**2**	**1**	**0.67**	1	2	0.34	1	2	0.34	0	3	nan	0	3	nan	0	3	nan

Sample	VM	NetMHC Score			NetMHCpan Score			NetMHCcons Score			MHCflurry Score			MHCnuggetsI Score			PickPocket Score		
							
		TP	FN	Recall	TP	FN	Recall	TP	FN	Recall	TP	FN	Recall	TP	FN	Recall	TP	FN	Recall

3812	0	^b^			^b^			^b^			^b^			^b^			^b^		
3942	2	0	2	nan	0	2	nan	0	2	nan	**1**	**1**	**0.50**	0	2	nan	0	2	nan
3948	0	^b^			^b^			^b^			^b^			^b^			^b^		
3978	0	^b^			^b^			^b^			^b^			^b^			^b^		
3995	3	**3**	**0**	**1.00**	2	1	0.67	2	1	0.67	1	2	0.34	2	1	0.67	2	1	0.67
4007	2	**2**	**0**	**1.00**	1	1	0.5	1	1	0.5	1	1	0.5	1	1	0.5	1	1	0.5
4032	3	0	3	nan	0	3	nan	0	3	nan	0	3	nan	0	3	nan	1	2	0.34

a Results indicated in bold are the best among the 12 methods. TP: true positives. FN: false negatives. VM: number of validated mutations.

b signifies that validation scores cannot be computed as the number of validated mutations provided by Tran *et al.* (2015) is 0.

### 3.3 Effects of cell population size on vaccine optimization

In this section we investigates how the cell population size influences the vaccine optimization.

**Figure 6.**
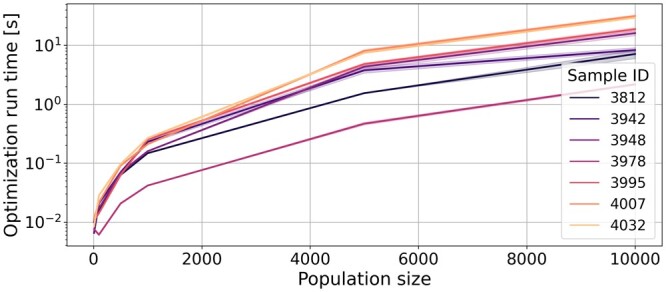
Vaccine optimization run time expressed as a function of the cell population size. The *y*-axis presents a logarithmic scale. Runtime is computed for the following cell population sizes: 10; 100; 500; 1000; 5000; 10 000. Results are averaged over 10 runs.

Figure 6 depicts how the optimization runtime varies as a function of the population size. A logarithmic scale is used for the y-axis. The run time is measured for the following population sizes: 10; 100; 500; 1000; 5000 and 10 000.

**Figure 7.**
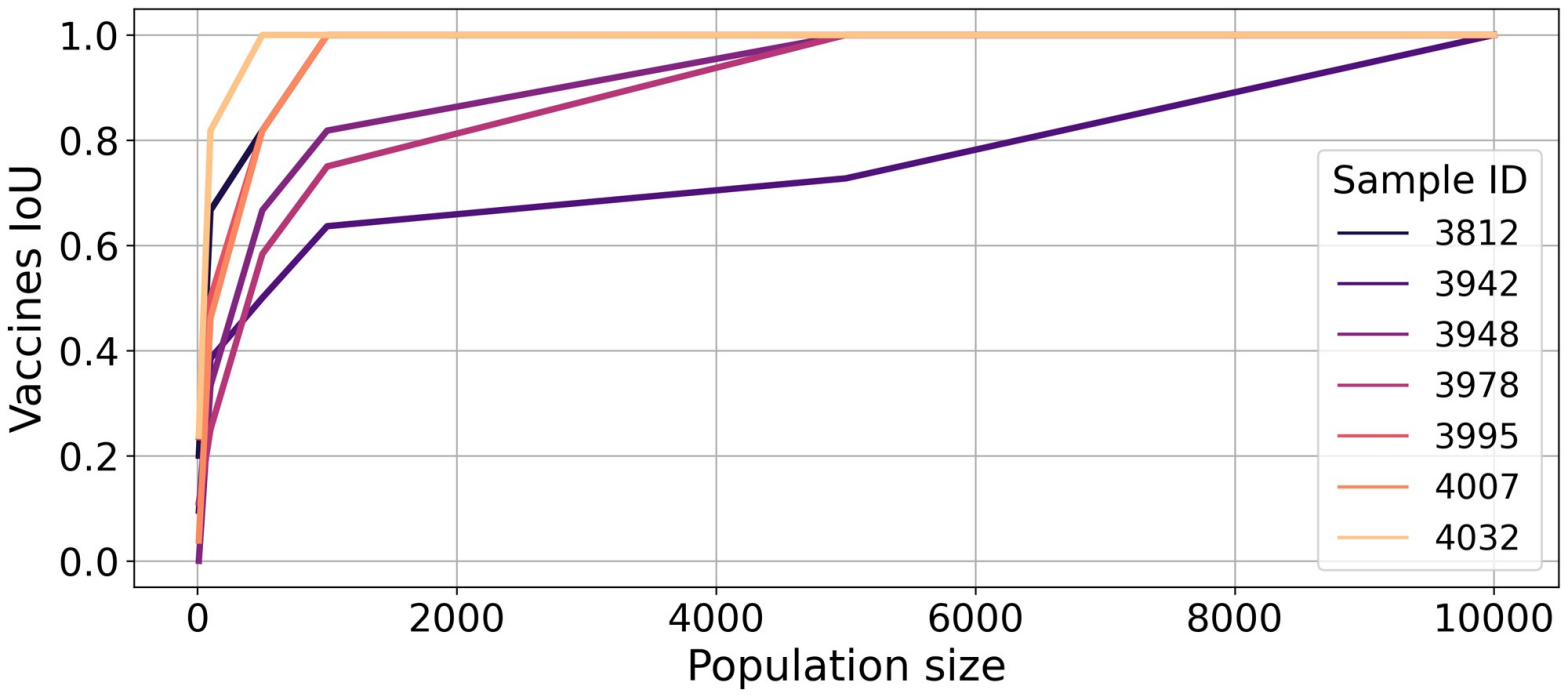
Intersection over union (IoU) of 10 different optimal vaccine compositions computed for different simulated cell populations. The plot depicts how the IoU varies as a function of the cell population size. Results are computed for the following cell population sizes: 10; 100; 500; 1000; 5000; 10 000.

For each considered population size, Figure 7 depicts the intersection over union (IoU) of 10 optimal vaccines computed for 10 different simulated cell populations. We compute the IoU as a measure of consensus across different optimal solutions: a high IoU implies low variability among different solutions of the optimization problem. We observe that as the population size increases, the IoU also increases. This implies that, as expected, bigger cell populations allow to better account for the stochasticity of the simulation. For most patient samples, the IoU saturates with a population size of 5000 cells, with an associated optimization run time ≤10 s.

## 4 Discussion

NeoAgDT is able to generate vaccines containing confirmed immunogenic neoantigens for all patients for which it was run. This is important as especially patients diagnosed with colorectal cancer can tremendously benefit from neoantigen cancer vaccines (Tran *et al.* 2015, Cafri *et al.* 2020, Picard *et al.* 2020). NeoAgDT shows improved recall based on experimentally confirmed neoantigens compared to more traditional, standard ranking approaches. In contrast to these standard ranking approaches, NeoAgDT explicitly simulates biological processes leading to cell surface presentation of neoantigens, resulting in a digital population of cancer cells. This simulation enables modeling competition during peptide-MHC binding, which is not taken into account by standard neoantigen identification approaches. Furthermore, NeoAgDT considers the optimal vaccine composition as the solution to an optimization problem defined by the vaccine budget, i.e. the number of elements in a vaccine. Implicitly, this encourages the selection of diverse neoantigens which cover the entire population of cells. In contrast, standard approaches may select high scoring yet redundant vaccine elements thus failing to cover the whole cell population. The cell population also allows to quantify estimated vaccine efficiency by counting the simulated cells presenting a neoantigen that is covered by the proposed vaccine composition. Our results showed that the selection of vaccine elements typically stabilizes across simulations at a population size of 5000 cells. The concept of vaccine budget can be extended by considering vaccine element length and amino acid composition, e.g. by applying a weight to each element. The output of NeoAgDT can be used to generate a vaccine construct, e.g. by using the pVACvector of pVACtools, which assembles a DNA vector avoiding junctional epitopes (Hundal *et al.* 2020). Regarding its input, NeoAgDT depends not only on the quality of predictions used as parameters in the cell simulation but also on the quality of sequencing data used to identify the tumor-specific mutations. We note, though, that *all* neoantigen identification methods crucially depend on the quality of this data. Mutation callers themselves vary substantially in terms of identified mutations. NeoAgDT can be run multiple times with different input mutations sets to characterize the impact of these differences. Alternatively, of course, an ensemble variant calling approach could be used (Koboldt 2020). The effect of specific prediction models can also be compared in a similar manner by running NeoAgDT multiple times using, e.g. different MHC binding predictors. NeoAgDT modular implementation allows the use of different sets of prediction algorithms, the omission of specific ones, or even the addition of further features for the cell simulation. This also makes it difficult to compare NeoAgDT to the neoantigen pipelines mentioned in the introduction; these tools function as a black box pipeline for several predictors and also usually require raw sequencing data as input. An alternative to pVACbind, which was used here, could be DeepImmuno (Li *et al.* 2021), which also features an elaborated immunogenicity score that could be used to replace the NetMHCstab prediction provided by pVACbind. It is crucial to emphasize that NeoAgDT also relies on the input from those predictive pipelines and its performance is dependent on the quality of predictions.

Currently, NeoAgDT simulates the MHC class I antigen presentation pathway; we focus on this pathway since pMHC recognition by CD8^+^ cells means immediate elimination of the cancer cell. For MHC class II, the effect of a response induced by CD4^+^ T cell recognition should be defined differently and is left for future work. A potential extension of NeoAgDT is accounting for allele-specific HLA expression during pre-processing (Anzar *et al.* 2022). Integrating RNA-specific mutations like splice variants is also possible if the respective input data type is available. Another improvement could be made by adding tumor clonality information, i.e. co-occurrence of mutations in cell populations belonging to the same clone. This could be integrated leveraging the output of tumor clonality prediction algorithms (Xiao *et al.* 2020, Abécassis *et al.* 2021). A more complex modification would be the simulation of the tumor microenvironment, especially T cell response. Diversity in T cell repertoire could be used as a proxy (Azizi *et al.* 2018, Han *et al.* 2020) or even prediction of neoantigen-specific T cell receptors (Montemurro *et al.* 2022, Grazioli *et al.* 2023), although this is currently not a feasible approach due to the limited reliability of current TCR binding prediction algorithms (Grazioli *et al.* 2022).

## Supplementary Material

btae205_Supplementary_Data

## Data Availability

RNA sequencing data from Tran *et al.* (2015) was downloaded from the Sequence Read Archive (SRA) under the accession number PRJNA342632, https://www.ncbi.nlm.nih.gov/bioproject/PRJNA342632.

## Code availability

Code is available with a noncommercial license under https://github.com/nec-research/neoagdt.
