# Supplementary material for: NeoAgDT: optimization of personal neoantigen vaccine composition by digital twin simulation of a cancer cell population
Source: Bioinformatics. 2024 Apr 13;40(5):btae205. doi: 10.1093/bioinformatics/btae205 (PMC11076149; doi:10.1093/bioinformatics/btae205)
Supplement: btae205_Supplementary_Data [file btae205_supplementary_data.docx]

# Supplementary information

## Description of input

The cell simulation requires five input files: 1) variant information including the variant allele frequency (VAF) or tumor and mutated read counts, 2) peptide sequences, i.e., all peptides containing at least one mutated amino acid, 3) gene expression, 4) HLA allele expression and 5) binding or prediction scores to be used in the simulation. All input files - except for expression – require the same mutation identifier, while expression files require the same gene identifiers as the variant file for mapping purposes. Any prediction scores have to be converted to values ranging from 0 to 1.0. Column and file names can be defined in the cell configuration yaml file, which also contains configuration options for the cell simulations. Example data is available to be used with the simulation, including all configuration files.

## Description of output

The output of the cancer cell population simulation contains the following header: repetition, cell_ids, presented_peptides, presented_hlas, simulation_name, mutation.

repetition specifies to which simulation a cell belongs to, since the user can operate multiple repeated simulations. cell_ids is a numeric identifier of the cell. presented_peptides and presented_hlas constitute a presented pMHC complex. simulation_name is a string identifier for the simulation. mutation specifies from which mutation the peptide is derived.

The optimization produces a first output file with the following header: peptide, repetition, simulation_name, weight.

Peptide specifies the neoantigens, or the neoepitopes selected by the optimization. Since we operate multiple cell simulations, repetition specifies to which one a row refers to. simulation_name is the same string identifier as specified above. Weight refers to the vaccine budget.

A second output file provides a final vaccine composition resulting from the aggregation of the various optimizations via a majority vote. Additionally, the optimization creates probability scores and plots to estimate the response probability of each cell.
